# A Distributed Model for Stressors Monitoring Based on Environmental Smart Sensors

**DOI:** 10.3390/s18061935

**Published:** 2018-06-14

**Authors:** Alberto de Ramón-Fernández, Daniel Ruiz-Fernández, Diego Marcos-Jorquera, Virgilio Gilart-Iglesias

**Affiliations:** Department of Computer Technology, University of Alicante, 03690 San Vicente del Raspeig, Alicante, Spain; aderamon@dtic.ua.es (A.d.R.-F.); dmarcos@dtic.ua.es (D.M.-J.); vgilart@dtic.ua.es (V.G.-I.)

**Keywords:** stress, environmental stressors, monitoring system, distributed sensors

## Abstract

Nowadays, in many countries, stress is becoming a problem that increasingly affects the health of people. Suffering stress continuously can lead to serious behavioral disorders such as anxiety or depression. Every person, in his daily routine, can face many factors which can contribute to increase his stress level. This paper describes a flexible and distributed model to monitor environmental variables associated with stress, which provides adaptability to any environment in an agile way. This model was designed to transform stress environmental variables in value added information (key stress indicator) and to provide it to external systems, in both proactive and reactive mode. Thus, this value-added information will assist organizations and users in a personalized way helping in the detection and prevention of acute stress cases. Our proposed model is supported by an architecture that achieves the features above mentioned, in addition to interoperability, robustness, scalability, autonomy, efficient, low cost and consumption, and information availability in real time. Finally, a prototype of the system was implemented, allowing the validation of the proposal in different environments at the University of Alicante.

## 1. Introduction

The growing popularity of the term “stress” makes clear the concern that provokes in our society nowadays. A search of the word “stress” using the search engine Google Scholar returns more results than other common terms such as “software” [1]. The relationship between stress and levels of biological substances such as cortisol, a hormone related to the human alertness, has also been studied [2,3]. 

We can find many definitions of the stress concept from different perspectives; sometimes, it leads to some confusion of the concept and mistaken beliefs. Thus, what is stress really? Answering this question is not easy, even for those considered as experts in this field. In fact, there is no single definition that agrees with the experts and stress and anxiety are often confused. The main difference is that, unlike stress, anxiety is considered as a disease by the medical community. In this research, we followed one of the most recent definitions of stress, by Roger in 1998, who described stress as a “preoccupation with the negative emotion following the event” [4]. The American Psychological Association (APA) classifies different types of stress according to their duration: acute stress, acute episodic stress and chronic stress [5]. Acute stress is the most common form of stress. Since it is short-term, acute stress does not cause such major damage as those found associated with long-term stress. When acute stress is repeated with relative frequency, it is known as acute episodic stress and if it persists over time it is known as chronic stress. This represents the type of stress most damaging to physical and mental health. It produces a prolonged wear over time which often causes the person to become accustomed to it and abandon the search for solutions. 

Regarding the impact on society, statistics show that stress levels have been increasing and it is affecting more people over the years. The events, agents and conditions that can cause stress to an organism, called stressors, increase daily. We can identify several types of stressors according to their origins: environmental stressors, daily stress events, life stressors, workplace stressors, chemical stressors and social stressors. In these stressors, we can identify two groups: the first one is made up of stressors that can be measured and quantified such as the environmental or chemical stressors; and, in the second group, we find the stressors that cannot be quantified such as the daily stress events. We focused this research on the stressors that can be quantified with the following objective: the more information we have about the stressors, the more control over stress we will have. 

From the sets of stressors previously presented, we have chosen the environmental stressors for two reasons. First, they belong to the group of stressors that can be measured so it is possible to set ranges of values for each stressor and associate them to a level of stress. Second, they do not depend on a person in a direct way; to explain that point we can think of a chemical stressor such as coffee and an environmental stressor such as air pollution: someone can decide not to take coffee but normally it is not possible to decide not to breathe polluted air. If we have information about environmental stressors (for example, what time a place is very noisy), we can adapt our behavior to reduce the effect of these stressors.

This paper describes a model and a specific monitoring architecture that integrates the sensing of variables that can affect our stress level. This way, we can be aware of external influences and control them to reduce our stress level. It is important to highlight that stress can depend on many factors which contribute in different ways for each person, so the system proposed in this work just give information about the variables monitored and when these variables can exceed predefined limits. For example, if loud noises affect a worker, the system can warn when the noise level is near the limit predefined or give information about what times are with the highest and lowest noise level.

Next, we present several works related to the stress monitoring, observing that most of the works are focused on stress evaluation in people, not on the monitoring system through which we can obtain information related to stressors. In Section 3, a general model to monitor variables from the user’s environment is presented. An architecture is defined in Section 4, considering the features of the model. In Section 5, we present the experimentation done with a prototype based on the proposed architecture. Finally, the conclusions and future work are drawn in Section 6. 

## 2. Related Work

Stress implies a physical and mental deterioration for those who suffer it and that can be spread out over time. That is why the management and control of this disorder help to prevent its symptoms from getting worse or being developed as a chronic behavior in the individual [5]. Stress assessment has traditionally been carried out from simple questionnaires related to life habits, feelings, experiences or future episodes [6,7]. Each answer has a score and the total score determines the level of stress. The main limitation of this method lies in the subjective evaluation of the answers. Although it is well known that certain traumatic experiences are intimately related to the increased level of stress or anxiety, the same experience can have a different impact on everyone. In contrast to the traditional way, the most novel ways of assessing stress seek to analyze physiological and behavioral variables. Stress causes physiological responses associated to heart rate, skin temperature, pupil diameter, electrodermal activity, etc. These changes can be measured using wearable sensors [8,9]. Moreover, different studies include artificial intelligence algorithms to classify and predict stressful situations from the physiological responses [10,11].

If we focus on stressors and, specifically, on environmental stressors, we can find studies on the relationship between stressors and illnesses. For example, Münzel et al. [12] analyzed the existence of a link between stressors, such as traffic noise and air pollution, and cardiovascular and metabolic diseases. It is also known that temperature and humidity can influence the development of some diseases [13]. For example, environments with high humidity promote the appearance of respiratory problems [14], bacterial infections [15] and sharpen bone diseases [16]. It is also proven that certain environmental stressors are closely linked with negative effects on psychological stress. For example, noise is a high-risk factor that can affect the well-being of people; in work environments, it negatively affects concentration and performance, increasing the stress level of workers [17,18]. Humidity is another stressor that affects the mental health of people. Shushtarian et al. [19] evaluated these effects. The results showed that workers who were exposed to higher humidity environments had higher stress levels compared to other workers. We can also find evidence that both indoor and outdoor crowded spaces have a negative effect on human behavior. The research developed by Park et al. [20] shows how the design of overcrowded urban environments, with a high level of noise, has negative effects on the health of people. In the same way, in [21], the findings suggest that the overcrowding of an indoor space can be considered a chronic source of stress.

Regarding the architecture of the systems used for the acquisition of stressors, an analysis of the literature on this field shows some examples of distributed systems designed for this purpose. In many cases, a distributed system is necessary due to the size of the area to be monitored or because the areas of interest are not together. As an example, the design of a distributed system to evaluate the noise level of an urban area is presented in [22]. Thanks to a network of interconnected sensors, it is possible to establish an acoustic map of the area. In the same way, in the work of Fuertes et al. [23], a distributed model for air pollution wireless monitoring in real time is developed, with the objective of detecting if the recommended contamination limits were exceeded in the analyzed areas. Finally, in [24], a distributed system is designed to control the temperature of the rooms in a residential building, keeping human thermal comfort. In addition to temperature, in [25], a remote monitoring system is developed to measure humidity through a distributed wireless network architecture of sensor nodes.

However, the aforementioned systems focus on the monitoring of an environmental variable for a specific purpose. None deal with the acquisition of environmental variables in a general manner, that is, without focusing on a particular variable. Furthermore, it is unusual to find a global system that monitors more than one variable at the same time. In this paper, we present a distributed model for the monitoring in real time of a general (non-specific) set of environmental variables that could affect the level of stress (stressors).

## 3. Proposed Model

The main objective of our proposal is the collection, analysis, storage, and provision of environmental indicators related to stress. This information is useful as a knowledge base for the development of systems, tools, or applications to analyze, solve, alert, or prevent situations of stress in humans.

As a starting point for the conceptualization of the work, we proposed the following as functional objectives to achieve a model: general, to support the monitoring in any type of scenario; flexible, without restrictions to monitor various types of environmental variables; proactive, notifying alerts for stressful situations; and high availability of information, so that all information collected can be consulted at any time and from any place.

**Figure 1:**
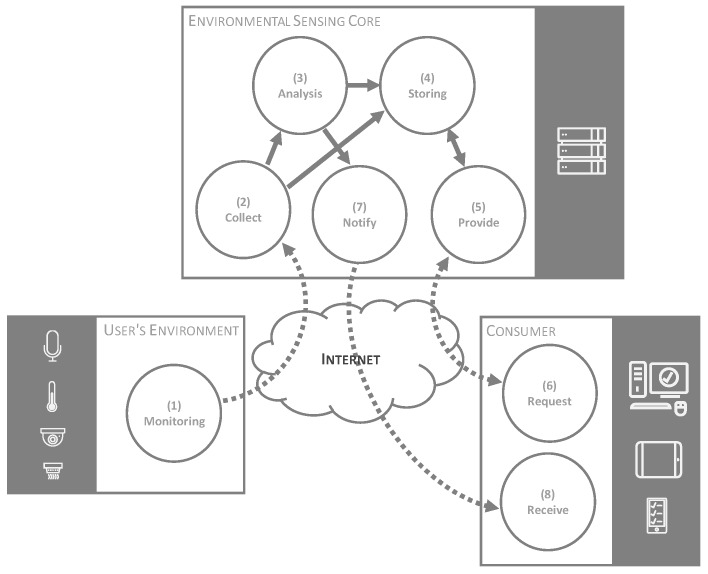
Proposed model.

Bearing in mind these objectives, to carry out the proposal, we defined a model where the functional processes that compose it are identified and decoupled (Figure 1). 

The first of these processes consists of a monitoring process (1 in Figure 1), where a set of environmental variables associated with stress is obtained through a sensing system located in the users’ environment. These variables can be, for example, the level of noise in the room, the flow of people moving through it or any type of environmental variable such as temperature, luminosity, or air quality. Variables are monitored by a set of sensors distributed around the work environment. It is in this process where the model incorporates a high degree of generality and flexibility, so that the model does not assume any distribution of the sensors, being able to integrate as many sensors as necessary.

The monitored data are collected, analyzed, and stored for later use (2–4 in Figure 1). In the analysis process, it is possible to contemplate both the historical evolution of each variable and the relationship between variables. This allows the generation of more valuable indicators of stress providing relevant and added value information to the final consumer of the system.

The information stored after the monitoring and analysis processes can be provided (5 in Figure 1) and requested (6 in Figure 1) at any time by consumer systems that require it. These consumers can be, for example, management systems for the control of the workers’ status, occupational risk prevention systems or personal systems that help users to detect and prevent stress situations. In addition, if during the analysis process certain values are detected in the indicators, the consumer systems can be notified (7 and 8 in Figure 1) proactively so that they carry out the appropriate actions. The notification process will be established by a previous configuration.

## 4. Architecture

**Figure 2:**
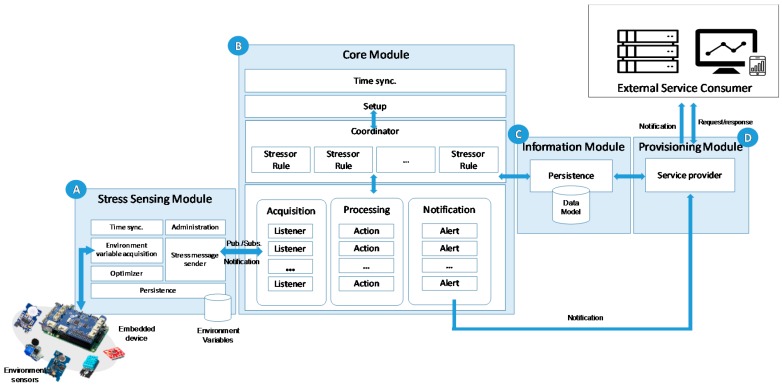
Architecture of the system.

**Figure 3:**
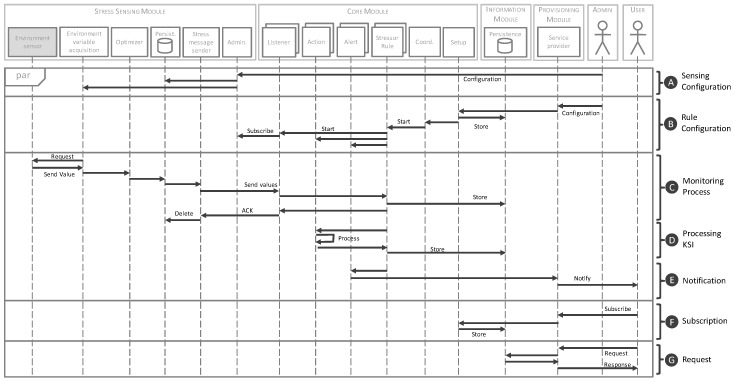
Sequence diagram of the main processes.

To support the described model, we proposed an architecture which is composed of four functional modules and whose general scenario is shown in Figure 2. Additionally, and to illustrate the system logic, Figure 3 shows a sequence diagram with the relationship and information flow between the modules.

The stress sensing module (A in Figure 2), such as a smart sensor, is composed of one or several sensors, which manages the environmental variable acquisition (stressors), and one small and low cost embedded device connected to the sensors that provides communication and computation features to record, process and send the information in an efficient and reliable way. The smart sensors are autonomous, decoupled and easily configured as a distributed system. This provide a high scalability and flexibility to the system, enabling its use in anyplace and anywhere.

**Figure 4:**
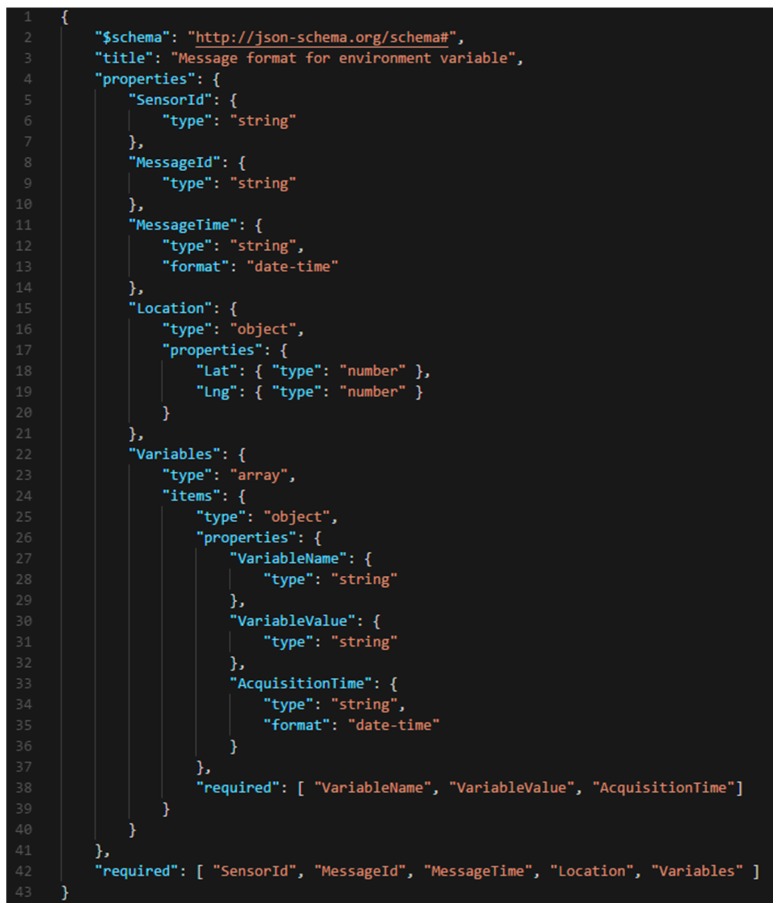
JSON schema for environmental variable message.

In the operation of the stress sensing module, first, the environment variable acquisition component is responsible for receiving environment values from a connected sensor (C in Figure 3). When the environment values are obtained, the optimizer component checks if the values have changed in reference to the last value sent. If a variable has changed, it is stored through the persistence component until it is delivered. The optimizer component provides an efficient communication with the core module, sending only the necessary information in every moment. Then, the value of the variable is sent to the core module through the stress message sender component. This component implements a notification pattern improving the efficiency of the system. In Figure 4, the JSON schema of a message is shown. In this schema, the fields that compose these messages are defined, as well as their type and features. It establishes a formal contract between sensors and the monitoring system, so that any new sensor that wants to integrate into the system should follow this schema. The core module will return an acknowledge message to confirm the information reception. This action involves the deletion of this information from the smart sensor through the persistence component. If there is a communication problem and the confirmation response does not arrive, the system has been implemented to send the information again when communication problems have been solved. Thus, the system guarantees the availability of information for further analysis. A fundamental aspect in any distributed system is the timing synchronization of the system components. In our proposal, it is a key feature because each obtained variable has a timestamp. In this sense, the time synchronize component guarantees the synchronization of each smart sensor of the system with the core module. Finally, an administration component is provided to configure the stress smart sensors (A in Figure 3).

The core module (B in Figure 2) is responsible for recording, processing and analyzing the information from smart sensors to transform it in value-added information. This information will be represented as a set of variables that we call Key Stress Indicators (KSI) and it is received through the acquisition component. Previously, this component must subscribe to each smart sensor distributed in the environment that we want to monitor, indicating which variables should be managed (B in Figure 3). The processing module is composed of a series of self-contained units named Actions that determine the transformation operations to calculate new KSIs. Each KSI will be associated with an action; this will allow the system to create as many calculated values as needed for decision-making, increasing the flexibility of the system. Each KSI can be a single atomic value obtained by the acquisition component, or a calculated value from a set of variables (D in Figure 3), and it is stored in the information module (C in Figure 2). 

After this process, KSIs are analyzed by the notification component through the corresponding alert unit (E in Figure 3). This component is responsible for warning the consumers previously subscribed (F in Figure 3). For this, each alert is defined by a rule composed of a set of conditions that establish, for example, a range of anomalous values. 

The flow done by the system components described previously is orchestrated through the coordination component by means of the stressor rules. Additionally, the setup component provides the necessary functionality to set the parameters of the modules: from the subscription process of the smart sensors to the KSI definitions and their warning ranges. 

The provisioning module (D in Figure 2) is responsible for providing the value-added information to external systems to help in the decision making in more complex systems. The module provides a service based on the principles of the SOA paradigm, implemented through the RESTful architectural style, and offering the resulting information through the JavaScript Object Notation (JSON) format. Achieving a model aligned with SOA principles (interoperability, discovery, well-defined contract, reusability, autonomy, composable, decoupling, coarse grained functionalities, and business-aligned), we get a set of benefits such as agility, integration, etc. 

The approach is geared toward the interaction between the service and external consumers. This interaction is implemented through an interoperable application programming interface (API). This interaction model implements two types of message exchange patterns. First, a reactive approach is based on the request–response pattern, when the consumer requests the necessary indicators for its operation and the monitoring service provides them (G in Figure 3). A second novel approach implements the proactive behavior of the stress monitoring service through a notification pattern based on publish–subscribe model, which complies with the SOA eventing principle (E and F in Figure 3). In this approach, consumers subscribe to the system to receive alerts associated to KSIs. This approach offers a more efficient and unattended monitoring model for the detection of specific, and previously configured, KSI ranges.

## 5. Experimental Environment and Results

To validate our proposal, a prototype was developed consisting of all the components described below. Subsequently, a set of experiments was carried out to validate the suitability and feasibility of our proposal.

### 5.1. Prototype

**Figure 5:**
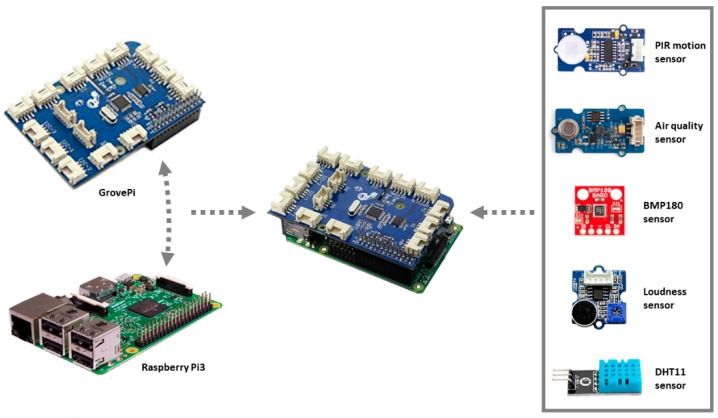
Hardware prototype components.

**Figure 6:**
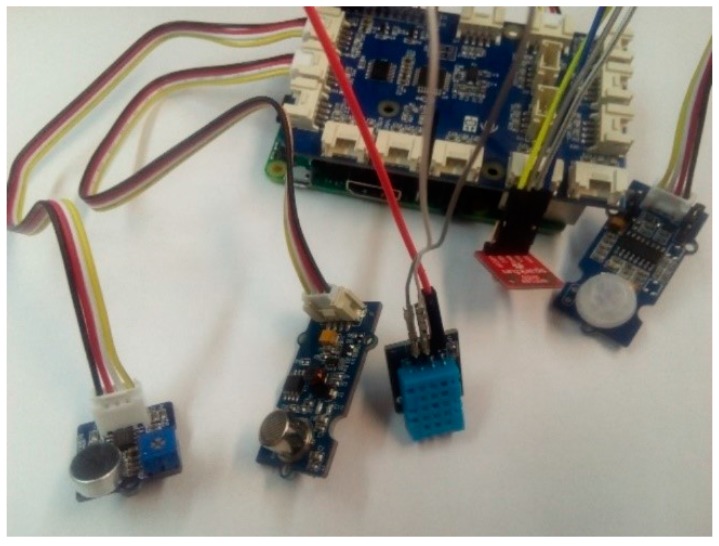
Hardware prototype implemented.

The sensing module (A in Figure 2) was developed using a small computer and five sensors. Figure 5 and Figure 6 show the components and the prototype implemented, respectively. Next, a brief description of all of them are provided:

- Raspberry PI v3. It is a low-consumption single board computer used as a hardware platform. Raspberry PI v3 can execute several processes simultaneously and in a robust way. It is also a flexible platform that allows the simultaneous connection of several sensors, thus being able to work in a centralized manner in case it is necessary due to the type of environment. The information collected is transmitted thanks to its wired and wireless network interfaces. Raspberry PI v3 is compatible with different operating systems, being Raspbian the one used in our architecture.
- GrovePi. To make easy the connection of the sensors, a GrovePi has been used. It is an add-on board with 15-4 pin ports (7 digital ports, 3 analog ports, 3 I2C ports and 2 serial ports) that couples to the Raspberry and that facilitates the connection of the sensors without the need of wiring them to the GPIO PinOut of the Raspberry PI.
- BMP180 sensor. It is a barometric pressure sensor, with high precision and extremely small consumption. Its measuring range is between 300 and 1100 hPa with a minimum margin of error of only 0.03 hPa. It also has a module for temperature and is designed to be connected directly to a small computer (Raspberry PI) through a two-cable I2C interface.
- DHT11 sensor. It is a digital temperature and humidity sensor. It has a capacitive humidity sensor and a thermistor to measure the surrounding air. It displays the data by means of a digital signal, with a range of measurement for the temperature from 0 to 50 °C and humidity from 20% to 90% of relative humidity. Similar to BMP180, this sensor also connects to the Raspberry PI through the I2C communication protocol.
- Grove-Loudness sensor. This sensor measures the existing sound in an environment. It amplifies and filters the recorded signal by a microphone embedded in the sensor and outputs an analog signal (0–1023). Its range of sensitivity is from −48 to 66 db. This sensor is easily connected through any digital port on the GrovePi.
- Grove-PIR motion sensor. This sensor allows to detect the movement by two slots sensitive to infrared radiation. The sensor translates any movement within its detection range into a positive output signal on its SIG pin. Easy to connect through any GrovePi digital port, it has a measuring range of up to 6 m, with a detection angle of 120°.
- Grove-Air quality sensor. It is a low power and cost-efficient sensor with high sensitivity. It has been designed to analyze the indoor air. It can detect a wide spectrum of harmful gases in the air such as alcohol, acetone, carbon monoxide, diluent, formaldehyde, etc. Although it does not provide quantitative data, it is a sufficiently valid sensor for obtaining qualitative results of air quality. Easy to connect to the GrovePi through any analog port, the output signal provides three levels of air quality: high pollution, low pollution and fresh air.

**Figure 7:**
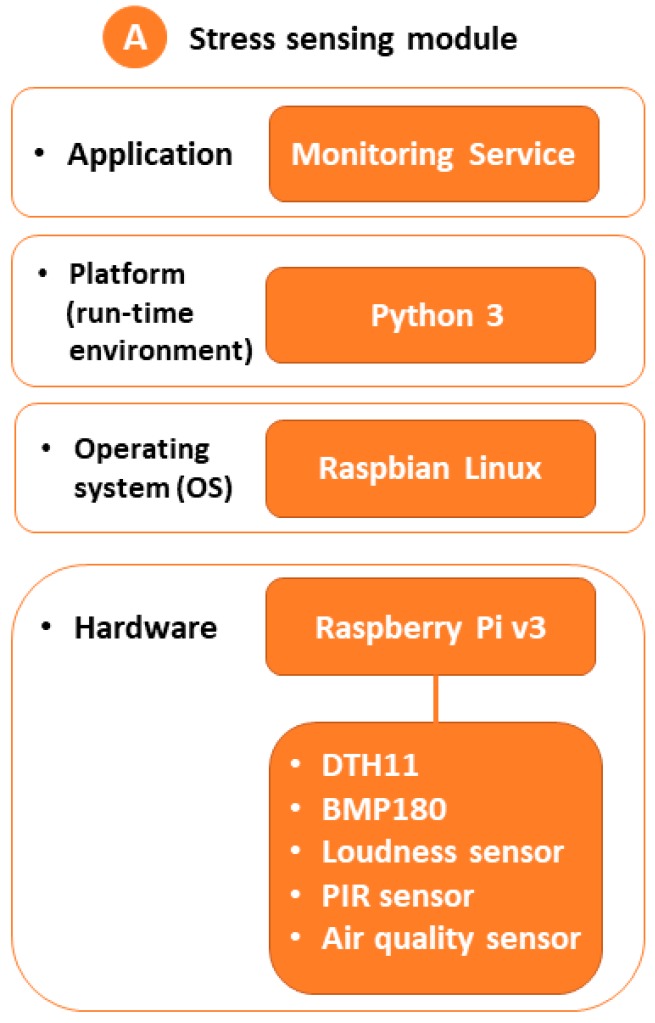
Architecture of stress sensing module.

For the software layers, Raspbian was used as the operating system and Python as the development platform, where the monitoring service has been implemented. Figure 7 summarizes the architecture of our prototype.

For the development of the Core, Information and Provisioning modules (B, C and D in Figure 2), NodeJS version 8.11.1 was used as a development platform. NodeJS is a modular and cross-platform runtime engine that allows the development of applications and services based on JavaScript language. As a database for the data model, MySQL Community Server version 5.7.21 was used, integrated into the project through the module for NodeJS mysql version 2.15.0. For the creation of the services, they were previously defined in RESTful API Modeling Language (RAML) and subsequently they have been implemented using the Express framework version 4.16.3.

**Figure 8:**
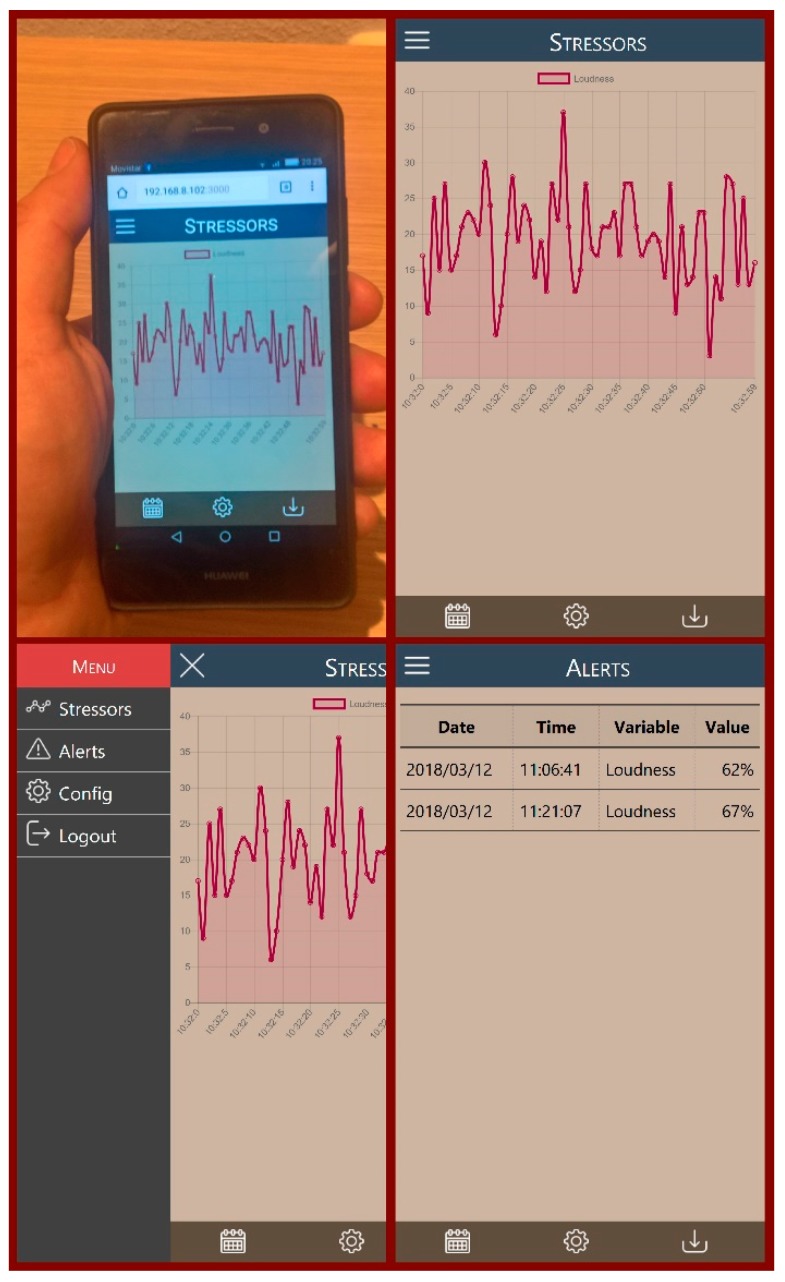
Web App prototype picture and screenshots.

Although the main objective of this work was the development of a system for the collection and provision of environmental stress indicators, to validate its correct use from external consumers, a web application was designed and developed that consumes the information obtained by our proposal. This application allowed validating the two basic consumption models: the information query, creating customized graphs of the monitored data during a period, and the collection and notification of alerts when, for example, a certain variable exceeds a predefined threshold. The application also allows configuring the parameters for monitoring and alerts management. For the development of the software the HTML 5, CSS 3 and JavaScript web standards and the Angular framework version 1.6.9. were used. The application implements a responsive interface that allows its use on computers, tablets or mobile phones (Figure 8).

### 5.2. Case Studies—Definition of Scenarios

The acquisition of data by the sensing module was carried out in two different scenarios within the Polytechnic School of the University of Alicante: the admission office and the restaurant. The admission office of the Polytechnic School is responsible for administrative tasks, such as supervising the proper management of the teaching resources, giving administrative attention to students, etc. This space is very similar to an office, with a fixed number of workers, and where there is a steady stream of students and teaching staff who come to solve administrative matters. For its part, the restaurant is a leisure space where students and university staff come together. It is a more relaxed environment, where, although there is a continuous flow of people throughout the day, the time of breakfast, lunch and dinner presents significant peaks of influx.

### 5.3. Experiments

Once the prototype was developed, a set of experiments was carried out in both scenarios to validate our proposal. These tests were aimed at checking the validity of the sensing module to collect data in real time in the proposed scenarios and the provisioning module to provide this information to the user.

**Figure 9:**
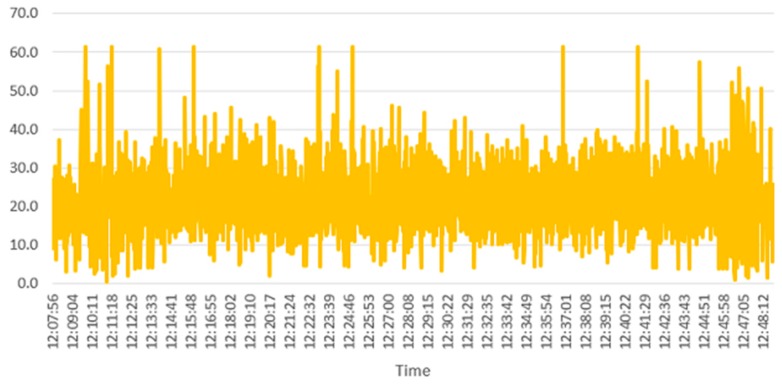
Evolution of the level of sound monitored by the sensing module in the restaurant.

**Figure 10:**
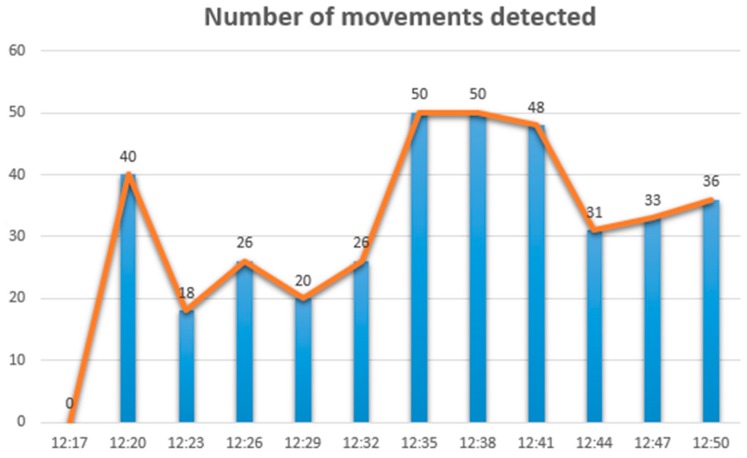
Increase of number of movements during a short interval in the restaurant.

Figure 9 and Figure 10 show an example of two stressors registered by our prototype during a brief time interval in the restaurant. This information can be consulted by users from third-party applications and help them to make decisions. For example, to avoid an environment that is not recommended due to its level of stress, a user may decide not to go to the restaurant at a certain time if the user considers that the noise level or number of movements detected is too high.

**Figure 11:**
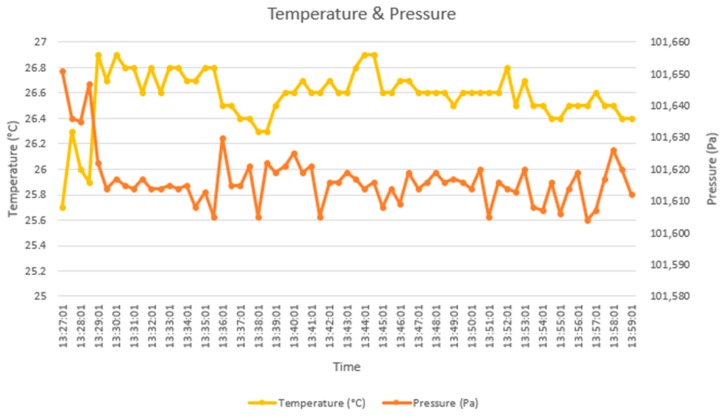
Evolution of temperature and pressure monitored by the sensing module prototype in the admission office.

Furthermore, thanks to the flexibility of our system, information can be obtained by comparing one or several stressors (see Figure 11) or by calculating new indicators from the original stressors.

The stressors collected in our experiment with the stress sensing module and the KSI calculated from the processing component are shown in Table 1.

**Figure 12:**
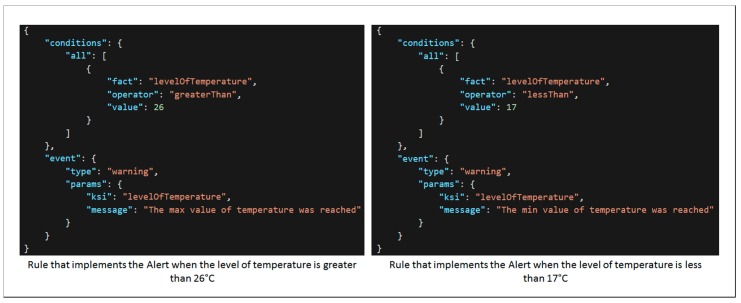
Example of two rules corresponding to alert units for level of temperature KSI.

In addition, these KSIs are analyzed by the corresponding alert unit from the notification component, which will warn the user about stressful situations. Alert units are rules implemented through a set of conditions using the NodeJS json-rules-engine library. An example of two rules for the level of temperature KSI is shown in Figure 12.

**Figure 13:**
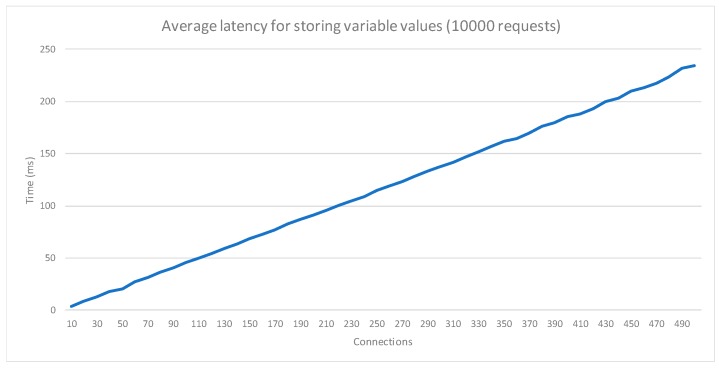
Latency for the acquisition module.

To validate the performance of the monitoring system, the latency in the variable acquisition module has been measured. The validation consisted of a set of tests where 10,000 storage requests were made, and where the concurrent access was increased from 10 to 500 in steps of 10 connections. The results (Figure 13) show that the service is stable, increasing the latency proportionally to the number of parallel connections. The response times for the requests are suitable for highly saturated environments, with numerous sensors registering values.

Experiments allowed validating a complete functional flow, from the monitoring of the indicators, to its consumption from an external application. Experiments were carried out in different scenarios, using various sensors, and configuring a set of KSIs to validate the generality and flexibility of the proposal. Likewise, the use of an embedded device as a platform for the development of smart sensors, in a service-based environment that allows its distribution in monitoring environments, has been validated.

## 6. Conclusions and Future Work

Stress is a health-related problem that can lead to serious illnesses. One way to tackle stress is to control the events, situations and factors that can provoke stress, the stressors. There is a set of stressors that can be measured and then, monitored. In this paper, we have presented a general model to monitor environmental stressors in a controlled place. We have defined an architecture to monitor this type of stressors using several connected sensors in a distributed sensing system. 

The proposal was tested in several workplaces at the University of Alicante obtaining information of variables such as noise, flow of people and air pollution. We also developed a web application that warns users about high levels of these stressors, so they can avoid these stressful environments or to take the necessary actions to reduce the effect of these stressors.

Technically, the experiments done show that the system developed can be used to analyze environmental stressors. We tested the system developed in two different scenarios, collecting different variables associated to environmental conditions which could be considered stressors. In the tests done, we could verify that the system accomplishes the features that we wanted to achieve. The generality and flexibility of features are especially important because, that way, our system can be used to monitor different variables without the need of using multiple, specific platforms for each variable we want to monitor. Furthermore, thanks to an architecture based on services, the distribution and scalability properties allow including more than one sensor at the same time and to register information of several variables simultaneously. This architecture also provides useful features to our proposal such as interoperability, proactivity and availability of information.

As future work, we plan collaborations with health professionals to use the model proposed as a knowledge base for research focused on stressors and how they can affect stress levels.

## Figures and Tables

**Table 1:** Stressors and tested KSIs.

Stressors	Key Stress Indicators
Noise	level of noise (%) max_value level of noise average level of noise
Air quality	level of air quality (ppm) max_value air quality
Humidity	level of humidity (%) ratio humidity/temperature
Tracking	number of movements ratio movements/min
Pressure	level of pressure (Pa)
Temperature	level of temperature (°C) max_value temperature
